# The Progress of Veterinary Science

**Published:** 1898-07

**Authors:** George Hilton

**Affiliations:** Winnipeg, Manitoba


### THE JOURNAL OF COMPARATIVE MEDICINE AND VETERINARY ARCHIVES.

VOL. XIX. *JULY, 1898.* No. 7.

### THE PROGRESS OF VETERINARY SCIENCE.[1]

By GEORGE HILTON, V.S.,

WINNIPEG, MANITOBA.

THE hand of time has fallen upon its endeavors to right the wrongs of past ages, and the dark doings of empiricism, superstition, and cruelty are beginning to yield to the influence of the lights of education, reason, and intelligence. Old prejudices are being wiped away, and the full value of the study of comparative medicine in its broadest sense is being better and better appreciated. Although it is a decided fact that veterinary science is still only in its infancy, its days have been far stretched, for we find its first steps coinciding with the medical profession in its very earliest periods, which are buried in the history of ancient Egypt and India.

It is well known that the ancient Egyptians owned and cared for the domestic animals, especially the ox, ass, goat, and goose, and evidence has also been obtained that antelopes were bred in immense numbers by these ancients; but sheep and other animals gradually displaced them as years passed on. Glancing over the customs of these people as far back as two thousand years before the Christian era, we find that animal property constituted their principal wealth. As they travelled through Asia, or gradually worked their way into Europe and Africa, accident and disease attacked their flocks, and attempts were at once made to save valuable property, and thus veterinary medicine began in a very insignificant and crude manner. As time wore on human medicine shared the devoted study of its ablest men; but their practical knowledge of anatomy had to be obtained from veterinary subjects, the times not permitting the dissection of human bodies, and thus from the

[1] Read before the Manitoba Veterinary Association, February 15, 1898.

beginning of the medical world the two branches of medicine were linked together. The years rolled on, and more and more attention was devoted to the study of the diseases of man, but veterinary medicine was relegated to an inferior position. This tendency increased until, as a result of certain influences, domestic animals became both valuable and indispensable, and as the numerous diseases swept them off by thousands, threatening health and interfering with the prosperity of the people, it became decidedly apparent that something should be done toward controlling the causes of such havoc. However, as the numbers in livestock increased without a corresponding increase in veterinary science, disease became widespread and raged through the numerous herds with unfailing rapidity and certain mortality. Affairs began to look serious in the extreme, and consequently investigations were persisted in by the ablest men the times could produce, for the health and wealth of the public were now at stake.

During the early days of the sixteenth century men who were endowed with the true veterinary ambition, summing up what little knowledge was available, placed it in books of agriculture. Thus the next step to success was attained, and veterinary literature was afloat on the realms of time. Although it was in a very unscientific manner, and not looked upon from the medical standpoint, it has proved to be with the polished touchings of time a most scientific and deep study. In fact, in those olden days, writings on veterinary medicine were to be found in large books on rural affairs. Books on horsemanship and the breeding and care of animals were also afloat, but a scientific treatise on veterinary medicine could not be found, with the single exception of the *Anatomy* of Carl Ruini. This work was issued in 1590, and formed the foundation of study for an independent science. Strange as it may appear, the other branches of our science developed so slowly that it was not until two hundred years later that works on the other fundamental branches, equal to the work of Ruini, were available.

However, sufficient progress was made during this early period to indicate that veterinary science was valuable, and as changed conditions developed in the habits of the people, in agriculture, transportation, and in warfare, the infectious diseases of animals became more widespread, causing more oppressive losses, and during the seventeenth and eighteenth centuries we find distemper, influenza, glanders, rinderpest, pleuro-pneumonia, foot-and-mouth disease, and anthrax raging in their varied localities with a determined severity, marking their victims along their course. During the last century, while all the communicable diseases prevailed more or less extensively, rinderpest was most prevalent and caused the greatest amount of distress and loss, passing through the southern provinces of Russia, Poland, and many of the States of Germany, Hungary, Austria, Italy, France, and Switzerland. It is stated that in 1740 the virus of this disease was so virulent that 200,000 head of cattle succumbed under its influence in Holland, while in Italy 70,000 head died in one year, and during the entire century this disease continued with greater or less severity, and we find it again between 1744 and 1746 robbing Holland of 200,000 more of its valuable livestock. Seemingly satisfied with its diastrous campaign in these countries, rinderpest broke out in Nottingham and Leicestershire, England, and carried off 40,000 cattle, while in Cheshire it wiped off 30,000 head in the short period of six months. Steps were now taken to check the progress of this notable plague, and in consequence 80,000 animals were slaughtered before this was accomplished. Foot-and-mouth disease was also prevalent during this period in France and other Continental countries, while anthrax rendered it almost impossible to rear livestock in many localities in Europe; glanders was prevalent in many districts. This terrible scouring of the country, by the loss of 200,000,000 cattle, meant a tremendous loss, amounting to $4,000,000,000, valuing the animals at $20 per head. Consequently, men of ability plunged deeper with prying inquisitiveness into the veterinary world.

Somewhere about the abatement of this disastrous season the stage-coach commenced to open up the country, and in a very short time became the fashion of the day. Military circles became more extensive, and horses naturally came largely in demand. Considering the unfortunate past, and the increasing demand for horses, the field now appeared ripe for the commencment of veterinary teaching, and in 1762 a Frenchman named Bourgelat opened the first school in Lyons. This was followed a year later by a second school at Charenton, near Paris, and still later by a third at Toulouse. These colleges were quickly patronized, and students were sent by all of the principal rulers. Unfortunately, veterinary literature was scarce, and the education imparted by these young schools did not satisfy the eye of the public. Many of the students proved themselves basely ignorant, and a gloom was cast over the profession.

Not long after this period a remarkable change occurred. The locomotive was introduced, and the old-fashioned globe seemed truly shaken by its intruding inhabitants. The result of such a stride was a question for the future. However, as it can be imagined, transportation increased steadily and rapidly, and the old world seemed to be lost in amazement. Under the old condition local wants were supplied entirely by local products, and what a locality did not produce was not consumed in that district. With the increased facilities for transportation the wants of the people became liberalized, at least as rapidly as the means for supplying them. Consequently a remarkable change commenced in social life, and especially in food products. It has been said, "that while meat is at least three times as expensive as it was sixty years ago, about three times as much per capita is used by the inhabitants of European countries," making it appear as though the degree of activity of a people is indicated by the amount of flesh consumed. All these changes have led to an astonishing growth of the live-stock industry, so that the public is far more dependent upon it now than it was in the last century, when the first veterinary schools were established. It appears as though the growth of veterinary science has been proportionate to the growth of the industry that is protected by it, and without such development in the veterinary world the livestock industry could not have attained its present proportions.

In the early decades of veterinary science it became quite evident that the schools then in existence were not thoroughly filling their functions. Therefore, after a sufficient number of well-trained veterinarians had developed, a thorough reorganization was accomplished, and the schools conducted under higher and more scientific principles, which was perfectly essential to bring the veterinarian to his path of duty.

The last century has, indeed, been a notable one. Reviewing its path, many are the flashes of genius that have illuminated the wide world of medicine, and have fixed for themselves stars in the firmament of science which will never cease to shine. As the years were added one by one to the past, they revealed to the minds of the investigators of science that many of the diseases of animals were communicable to man, and it has gradually become the duty of the veterinarian to guard against the first appearance of any contagious disease, both for the sake of the animals and that of the human race. On the other hand, there has been discovered the protective action of some diseases of animals on the human system, the one most widely known being vaccinia against smallpox and the antidiphtheritic serum prepared from the blood of the noblest of quadrupeds. The question as to how these act is one of the most interesting problems of modern science, and gives plenty of scope for a future genius. With this decade has also come the general acceptance of the bacterial origin of disease, and no question of medicine in late years has occupied so much space and thought, or caused so much scientific research and experiment as the origin, development, and communication of disease. We to-day have a knowledge, though yet limited, of organisms infinitely small, the existence of which was not suspected by the ordinary practitioner till within the last few years. We have a faint idea of the great good done by Bonnet, who, between 1740 and 1793, advanced the theory of universal dissemination of seeds and spores, known as the pre-existing germ-theory. (This is the oldest and probably the most widely-accepted theory ever known in the history of medical science.) During the same period Needham came to the conclusion that infusion of organic matter in hermetically closed vessels, after boiling, brought forth minute organisms, which is known as the vegetative or producing-force theory. The method of study introduced by Needham forms the basis of all subsequent experiments on this question, as well as the culture researches in connection with the organisms of disease. Dr. Budd, in 1849, claimed the body was invaded by organisms, which, under certain conditions and surroundings, caused infectious diseases; but it is only of late years—in fact, since 1870—that we have been shown the relation of microörganisms to those diseases which most closely interest the veterinarian, as anthrax, septicæmia, cholera in its various forms, glanders, rabies, pleuro-pneumonia, and the greatest of all scourges, tuberculosis, which Koch and Pasteur have so enlightened in past years. The satisfying of all the requirements essential to prove a germ causative of a disease is difficult, and has led to what may be termed a special branch of the medical art. This theory, with the aid of veterinary science, is gradually bringing to light how much of the physical disease and death of man is due to direct transmission from corresponding disease in our domestic animals. Again, looking into the kingdom of parasites, we find some of the most deadly of man's tormentors coming directly from our livestock. Trichinia, echinococcus, cysticercus bovis and actinomycosis may be mentioned in this connection.

The more intimately we acquaint ourselves with the subject of communicable or contagious disease the more deeply are we impressed with the fact that there is the closest relationship and interdependence between these affections as they appear in man and animals. In fact, in many cases, as in the echinococcus, and even the trichinia, the successive appearance of man and animal as the hosts of the parasite, at the different stages of its development, is a condition of its propagation, and, so far as known at present, it is a matter of impossibility for these parasites to live out their existence in one host. In the case of contagious affections due to microbes the same alternation from man to beast is not so essential to their maintenance, and yet the intimacy of the relation between the domesticated animal and the civilized man is so close that many such diseases are largely propagated in this way. Thus glanders and anthrax stand out largely as industrial diseases.

Of late years the general public has been more exercised over tuberculosis than any other complaint which is common to man and beast. There is doubtless good reason for this. This white plague of the north, by far the most deadly affection of man, has also been the most prevalent chronic disease of our dairy herds, and its extension in the human race bears a remarkable ratio to the utilization of the bovine races for dairy products and beef, and concurrent testimony obtained on so large a scale, and from such widely different sources, is not to be lightly set aside. The very latency of the disease in certain systems, and the absence of all prominent outward manifestations of illness, are potent factors in the propagation of the infection. A disease that is quickly fatal, like smallpox, yellow fever, or cholera in man; or anthrax, rinderpest, and Texas fever in cattle, is easily dealt with, since, whenever the germ exists in connection with susceptible subjects, its presence will be speedily manifested and can at once be circumscribed and crushed out. These make their attack in broad daylight, and we are warned to fortify every pass and strengthen every defence. But the stealthy tuberculosis, which glides up in the darkness and silence, and, as it were, saps our walls of defence without visible manifestations or audible sound, and suddenly appears, when least expected, in the interior of our most trusted keep, is by far the most dangerous enemy.

As bacteriologists we recognize incompatibilities and antagonisms between the living cells of the animal body and their products, on the one hand, and the pathogenic microbe and its products, on the other. How far can we avail of these to strike a balance favorable for and protective to the animals? We are as yet on the mere confines of this great science of bacteriology. In the vast microscopic world, full of attractions and repulsions of living cells and microbes, or neutralizations and physiological antagonisms, of leucomaïns, ptomaïns, toxins, and enzymes, there are many and bright promises for the future of preventive and therapeutic medicine. Without undue arrogance, it may be asserted that to the veterinarian has been allotted a large measure of responsibility in relation to this work, and we find at the present time veterinary science abreast with the times. Let us consider for a moment what good has been accomplished by the veterinary schools and what effect their work has had on national life and prosperity.

Unfortunately, the most effective work has been in the direction of controlling the infectious disease of animals. Rinderpest has been exterminated from Europe, and does not prevail anywhere extensively, excepting in South Africa, where it has been numbering its victims by thousands. Contagious pleuro-pneumonia has been controlled to such an extent in Europe that it does not cause serious losses at this time. Anthrax has been studied with such success that an efficient vaccine has been discovered, by means of which animals can be inoculated, and the development of the disease prevented. Moreover, so much light has been thrown on the life-history of the germ that we now know what measures must be taken to prevent the spread of this disease or its establishment in a certain locality after it has been introduced. Foot- and mouth-disease, although it still occurs in most of the Continental countries, has succumbed to the veterinary police measures that are now enforced to such an extent that it has not caused serious loss for a considerable number of years. Mange of horses and scab of sheep, diseases that were formerly dreaded so acutely by horsemen and shepherds, are now comparatively rare. Distemper and glanders of horses occur from time to time, but are kept in check so well that they no longer seriously menace the horse-breeding industry. Texas fever, a disease which formerly destroyed thousands of cattle every year in the United States, has been studied so thoroughly, and preventive measures taken, that outbreaks of Texas fever are practically unknown north of the "Texas fever line." This can also be looked upon as one of the triumphs in veterinary medicine. Hog-cholera is a disease upon which a good deal of light has been shed during the past few years, but we are still without an effective means of preventing its ravages. Tuberculosis, again, is an affection that is arousing much discussion and occasioning extensive losses, and leaves a wide field open for future investigations.

The discoveries in serum-therapy are of equal interest and importance to the physician and veterinarian, and much of this recent development results from the work of distinguished members of our profession. The international trade in livestock has developed enormously during the past few years. It is a comparatively small matter to ship cattle three, four, or five thousand miles, and, with modern facilities, these journeys can be made in a very few days. International trade in livestock and livestock products, such as hides, skins, and wool, has also reached immense proportions. These also are carried quickly between points on opposite sides of the world. It will thus be seen that without proper supervision it would not be difficult for the most dangerous diseases to be carried long distances from the most remote countries into previously uninfected territory. But every civilized country has a force of efficient veterinarians, and a more or less perfect system of quarantine and control, by means of which the ravages of disease that would otherwise be conveyed by this international commerce are avoided.

In its relation to public health, veterinary science is growing in importance; the meat- and milk-supply of large cities is now coming under the supervision of the veterinarian.

The relation of the veterinarian to the improvement of livestock, through the introduction of improved breeds and species, and improvement by care and selection, is a very important one.

In conclusion, allow me to turn to another channel—"the turf." Here we find a tremendous change, due to the applied skill and teachings of the veterinary art. In 1818 the first trotting performance took place in America, when a horse by the name of Boston Blue was driven a mile in three minutes. Later, in 1825, the New York Trotting-club held its first meet, with three entries under the saddle—Screw, Screwdriver, and Betsy Baker. Time, first heat, 5.36; second, 5.38, being at the rate of 2.50. As time passed on greater interest was taken by the sporting community, and the numbers and quality of the race-horse gradually increased, and coming to 1859 we find the world astonished by Flora Temple's performance in 2.19¾. As the news flashed over the country the opinion was freely expressed that the limit of speed in the harness-horse had been reached. Eight long years proved this erroneous, when, in 1867, Dexter performed it in 2.17¼ at Buffalo. He was king for four years, when in turn he was dethroned by Goldsmith Maid, who, in 1871, trotted the mile in 2.17, reducing it to 2.16¾ the following year, and still, two years later, in a trial against time, she lowered it to 2.14. From this on the race-horse has gradually increased in speed and quality, and at present we find Star Pointer as king of pacers, with the remarkable record of 1.59¼, while Salvator holds his place among runners with the mark of 1.35½, thus showing a wonderful result of the achievement of skill.

Lastly, we find that we are the members of a profession that is evidently destined to take the foremost rank in the science of medicine. Remembering it is the men who give the dignity to the profession, and not the profession to the men, it behooves us to bear ourselves in our relations to others in a manner that will always throw upon our profession the utmost honor and respect.

